# Apoptosis-Like Death in Bacteria Induced by HAMLET, a Human Milk Lipid-Protein Complex

**DOI:** 10.1371/journal.pone.0017717

**Published:** 2011-03-10

**Authors:** Anders P. Hakansson, Hazeline Roche-Hakansson, Ann-Kristin Mossberg, Catharina Svanborg

**Affiliations:** 1 Department of Microbiology and Immunology, University at Buffalo, State University of New York, Buffalo, New York, United States of America; 2 The Witebsky Center for Microbial Pathogenesis and Immunology, University at Buffalo, State University of New York, Buffalo, New York, United States of America; 3 New York State Center of Excellence in Bioinformatics and Life Sciences, Buffalo, New York, United States of America; 4 The Section of Microbiology, Immunology and Glycobiology (MIG), Department of Laboratory Medicine, Lund University, Lund, Sweden; 5 Singapore Immunology Network, A*STAR, Singapore, Singapore; Institut de Pharmacologie et de Biologie Structurale, France

## Abstract

**Background:**

Apoptosis is the primary means for eliminating unwanted cells in multicellular organisms in order to preserve tissue homeostasis and function. It is characterized by distinct changes in the morphology of the dying cell that are orchestrated by a series of discrete biochemical events. Although there is evidence of primitive forms of programmed cell death also in prokaryotes, no information is available to suggest that prokaryotic death displays mechanistic similarities to the highly regulated programmed death of eukaryotic cells. In this study we compared the characteristics of tumor and bacterial cell death induced by HAMLET, a human milk complex of alpha-lactalbumin and oleic acid.

**Methodology/Principal Findings:**

We show that HAMLET-treated bacteria undergo cell death with mechanistic and morphologic similarities to apoptotic death of tumor cells. In Jurkat cells and *Streptococcus pneumoniae* death was accompanied by apoptosis-like morphology such as cell shrinkage, DNA condensation, and DNA degradation into high molecular weight fragments of similar sizes, detected by field inverse gel electrophoresis. HAMLET was internalized into tumor cells and associated with mitochondria, causing a rapid depolarization of the mitochondrial membrane and bound to and induced depolarization of the pneumococcal membrane with similar kinetic and magnitude as in mitochondria. Membrane depolarization in both systems required calcium transport, and both tumor cells and bacteria were found to require serine protease activity (but not caspase activity) to execute cell death.

**Conclusions/Significance:**

Our results suggest that many of the morphological changes and biochemical responses associated with apoptosis are present in prokaryotes. Identifying the mechanisms of bacterial cell death has the potential to reveal novel targets for future antimicrobial therapy and to further our understanding of core activation mechanisms of cell death in eukaryote cells.

## Introduction

During our studies of the antimicrobial activity of human milk, we identified a complex of alpha-lactalbumin (ALA) and oleic acid that induces apoptosis in tumor cells, without affecting healthy, differentiated cells [1], [2]. The same complex showed strong bactericidal activity against specific pathogens of the oral cavity and respiratory tract, with the highest activity against the gram-positive organism *Streptococcus pneumoniae* [3]. In the complex, designated HAMLET for “human alpha-lactalbumin made lethal to tumor cells”, alpha-lactalbumin is present in a partially unfolded conformation that is stabilized under physiological conditions by C18:*n* cis unsaturated fatty acids, the most prevalent fatty acids in human milk [2], [4]. The native, folded form of ALA, with lactose synthase activity, has no tumoricidal or bactericidal effect, however [1], [3].

Programmed cell death or apoptosis in eukaryotes is executed by the consecutive activation of specific biochemical pathways that produce a dying cell, with typical morphology, such as cell shrinkage, membrane blebbing, chromatin condensation, as well as distinct DNA fragmentation [5]. This type of programmed cell death represents an important mechanism to regulate tissue function and homeostasis in multicellular organisms [6] but is also used by unicellular eukaryotes to regulate their optimal adaptation to their environment [7]. Although, primitive forms of programmed cell death and terminal differentiation have been described in prokaryotes also [8], there has been no information to date to suggest that bacterial death show similarities to eukaryote apoptosis.

In this study, we demonstrate that HAMLET triggers DNA fragmentation, as well as morphological and biochemical changes in *S. pneumoniae,* resembling apoptosis in tumor cells. We also describe similarities in the responses to HAMLET between mitochondria and bacteria. Our studies suggest for the first time that bacteria contain basic cell death programs that are similar to those involved in eukaryotic cell apoptosis.

## Results

### 1. HAMLET kills tumor cells and bacteria

**Figure 1:**
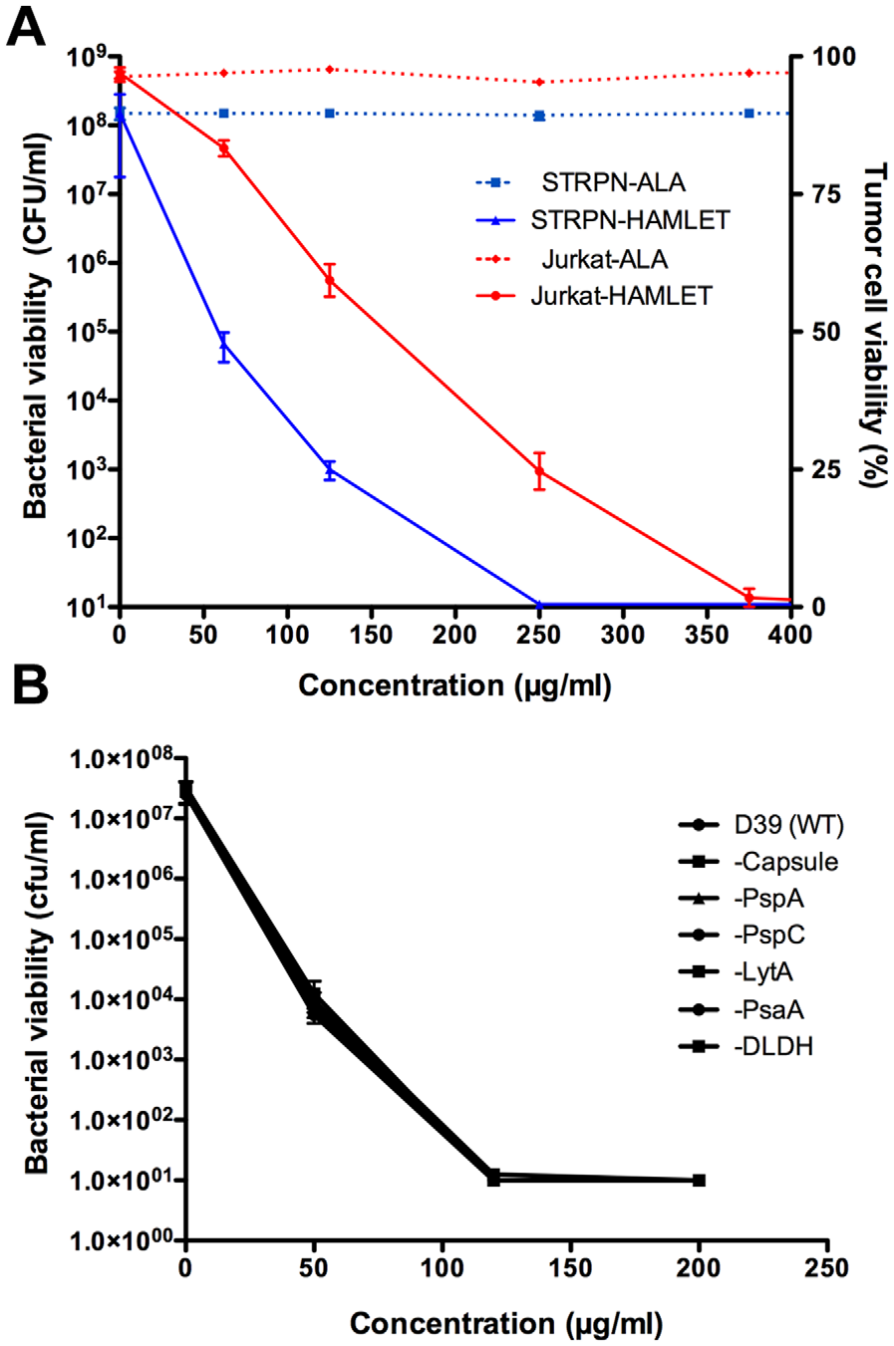
Bacterial and tumor cell death induced by HAMLET. A) Jurkat cells and *S. pneumoniae* D39 were incubated with increasing concentrations of HAMLET or human alpha-lactalbumin (ALA) and viability was monitored after 6 h or 1 h of incubation for Jurkat and bacterial cells, respectively. Viability of Jurkat cells are presented on the right Y axis in per cent viable cells in the suspension as determined by trypan blue staining and viability of bacteria are presented on the left Y axis as colony forming units (CFUs) per ml suspension (detection limit in the assay was 10^1^ CFU/ml). ALA (hatched lines) did not kill any of the organisms whereas HAMLET (solid lines) killed both Jurkat and bacterial cells in a dose-dependent manner. The data represent the mean of three individual experiments with standard deviation error bars. B) Role of bacterial virulence factors in HAMLET-induced killing of *S. pneumoniae.* Pneumococcal strains lacking capsule, Pneumococcal surface proteins A and C (PspA and PspC), autolysin (LytA), pneumococcal surface adhesin A (PsaA), or dihydrolipoamide dehydrogenase (DLDH), all associated with virulence, were treated with 50, 120 and 200 µg/ml of HAMLET for 1 hour at 37°C and viable organisms were determined by plating organisms on solid agar and counting colony forming units after overnight growth. The graph depicts the mean of three experiments. There was no significant difference in sensitivity related to lack of these virulence factors.

Cell death in response to HAMLET was quantified in parallel in Jurkat leukemia cells and *S. pneumoniae* (Fig. 1A). HAMLET killed Jurkat leukemia cells in a dose-dependent manner with 50% death occurring at a concentration of 200 µg/ml after 6 hours. At the same concentration, HAMLET reduced the viability of *S. pneumoniae* by more than 6 log_10_ within 1 hour, with complete eradication of the inoculum (8 log_10_) obtained at 250 µg/ml (Fig. 1A). These concentrations are within the physiological range, as the concentration of ALA is especially high (1,000–2,000 µg/ml) in human milk [9]. The effect appeared to be general among pneumococci, as over 25 pneumococcal strains of nine different capsule types were equally sensitive to HAMLET-induced death (Table 1). In accordance with earlier results [2], [3] the calcium bound holo-form of ALA had no tumoricidal or bactericidal activity even at concentrations up to 10,000 µg/ml (Fig. 1A).

**Table 1:** Strains of *S. pneumoniae* tested for HAMLET-sensitivity.

Strain name	Capsule type	Bacterial viability (CFU/ml)		
		Untreated	HAMLET62 µg/ml	HAMLET125 µg/ml
L82006	1	2×10^8^	9×10^4^	8×10^2^
D39	2	3×10^8^	3×10^4^	5×10^2^
DBL2	2	9×10^7^	1×10^5^	7×10^2^
WU2	3	2×10^8^	7×10^3^	<10
A66	3	2×10^8^	4×10^4^	8×10^2^
3JYP2670	3	1×10^8^	7×10^4^	4×10^2^
ATCC 6303	3	1×10^8^	2×10^4^	9×10^2^
EF10197	3	3×10^8^	1×10^5^	2×10^3^
TIGR4	4	5×10^8^	8×10^4^	5×10^2^
EF3296	4	5×10^8^	6×10^4^	3×10^2^
L81905	4	8×10^7^	2×10^4^	8×10^2^
DBL5	5	2×10^8^	3×10^4^	7×10^2^
BG9273	6A	4×10^8^	5×10^4^	1×10^3^
BG9163	6B	5×10^8^	3×10^4^	6×10^2^
BG7322	6B	3×10^8^	1×10^4^	4×10^2^
L82016	6B	3×10^8^	2×10^3^	<10
BG30-11	6B	2×10^8^	9×10^4^	5×10^2^
EF3559	14	3×10^8^	1×10^5^	8×10^2^
EF1488	15A	3×10^8^	6×10^4^	4×10^2^
L82013	19F	3×10^8^	8×10^3^	<10
EF3030	19F	5×10^8^	1×10^4^	<10
EF10175	19F	4×10^8^	7×10^3^	<10
ATCC 49619	19F	1×10^8^	4×10^4^	2×10^2^
BG8826	23F	9×10^7^	9×10^3^	<10

To examine if death in response to HAMLET was modified by the virulence of the *S. pneumoniae* strain, we used the wild-type strain D39 and deletion mutants lacking each of five virulence-associated pneumococcal surface molecules (capsule, pneumococcal surface proteins A or C, pneumococcal surface adhesin A, and dihydrolipoamide dehydrogenase [10]–[14]. These molecules are known to interact with host targets, including some molecules present in human milk and to bind and inactivate bactericidal host defense molecules such as complement proteins and lactoferrin. Wild type or mutant strains were exposed to HAMLET and the loss of viability was quantified by viable counts. No differences were recorded, suggesting that these molecules are not involved in HAMLET's bactericidal activity (Fig. 1B).

**Figure 2:**
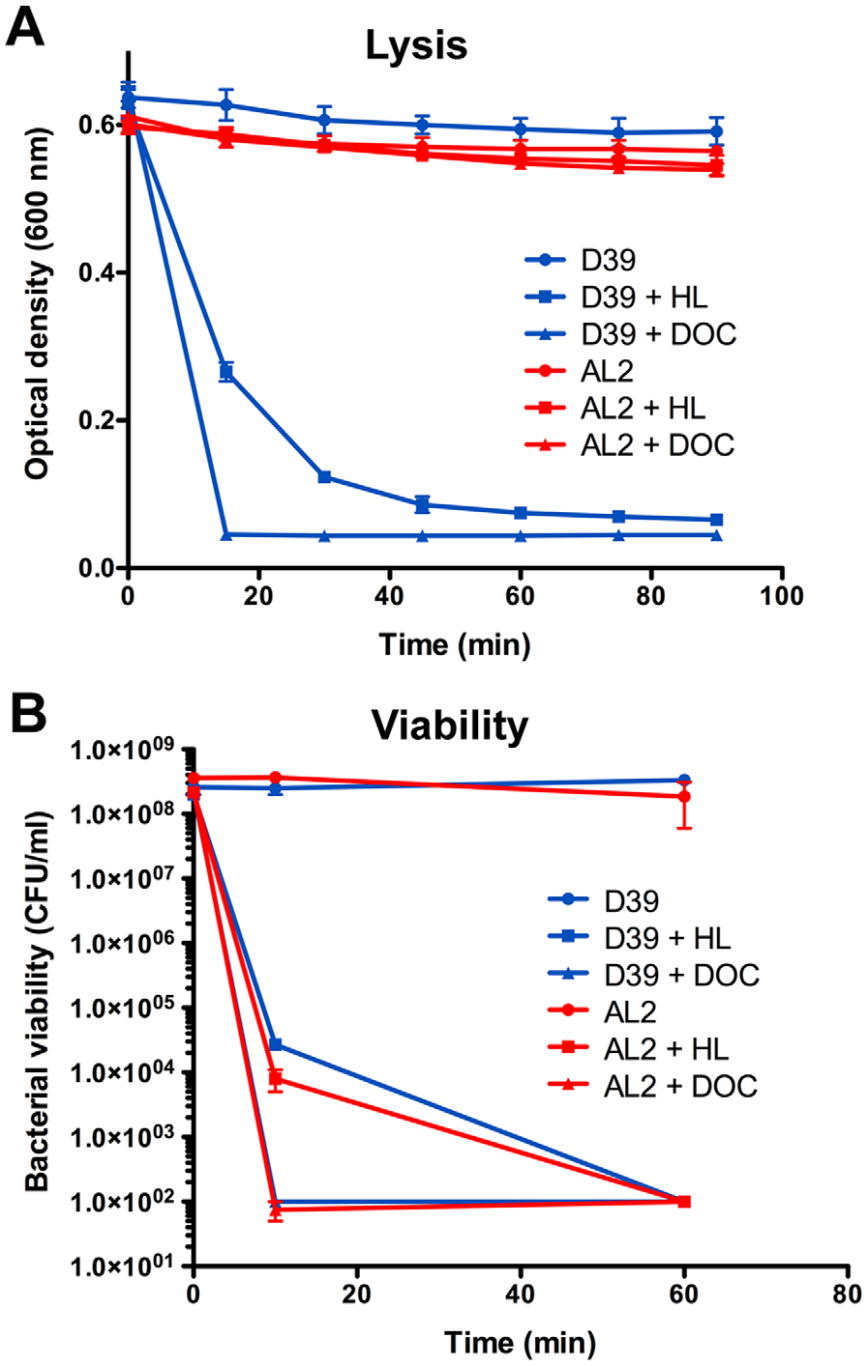
Role of autolysin in HAMLET-induced lysis and killing of *S. pneumoniae.* Pneumococcal strains D39 and AL2 (D39 **Δ** *lytA*) were treated with 150 µg/ml of HAMLET (HL) or 0.1% of the bile salt deoxycholate (DOC) over 90 minutes. As pneumococci are bile salt sensitive streptococci and lysis from bile salts is LytA dependent, deoxycholate was added as a LytA-dependent control. *A*) The optical density of the suspension was monitored at 600 nm every 15 minutes to assess the lysis of bacteria. *B*) After 10 minutes and 60 minutes bacteria were serially diluted and plated on blood agar overnight to determine viable colony forming units (detection limit in the assay was 10^2^ CFU/ml). The data represent the mean of three individual experiments with standard deviation error bars. HAMLET and DOC killed both strains equally well, but lysis was only observed in the autolysin positive D39 strain.

HAMLET-induced killing of *S. pneumoniae* was accompanied by lysis of the bacterial cells, detected by a parallel decrease in viability and turbidity of the bacterial suspension (Fig. 2A). To address the mechanism of bacterial lysis, autolysin-negative mutants in *S. pneumoniae* D39 were exposed to HAMLET. Bacterial lysis was autolysin (LytA)-dependent but LytA-negative bacteria were killed as efficiently as wild type bacteria (Fig. 2B), indicating that lysis was independent and occurred downstream of the initiation of HAMLET-induced death. In this regard, HAMLET's activity was similar to that of the well-characterized bile salt deoxycholate (DOC), which was used as a positive control [15].

### 2. Apoptosis-like morphology of HAMLET-killed pneumococci

To examine if HAMLET interacts with analogous targets in tumor cells and bacteria we first examined apoptosis-associated phenotypes in A549 carcinoma cells and pneumococci treated with HAMLET. Chromatin condensation and DNA fragmentation, two morphological hallmarks of apoptosis, were investigated by microscopy and gel electrophoresis, respectively.

**Figure 3:**
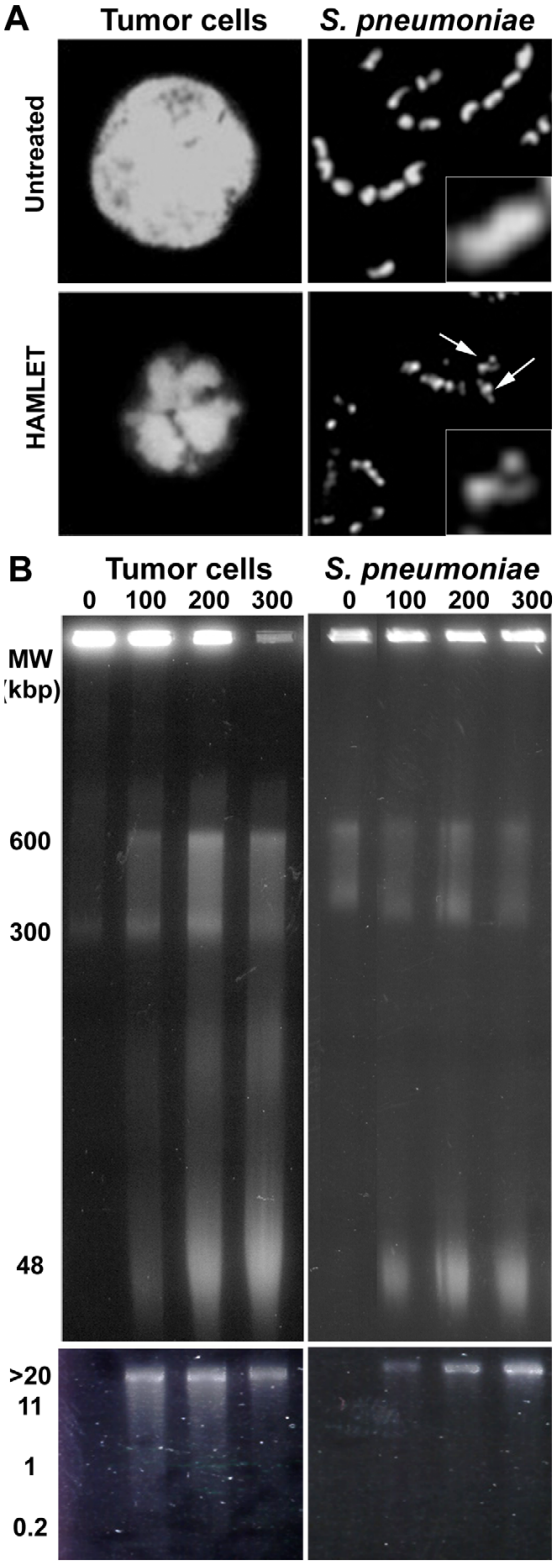
Chromatin condensation and fragmentation induced by HAMLET in tumor cells and *S. pneumoniae*. *A*) Chromatin morphology in HAMLET-treated A549 carcinoma cells and *S. pneumoniae* AL2 (D39 **Δ** *lytA*) cells after 6 h and 1 h of incubation, respectively. All cells were fixed in 4% paraformaldehyde solution and stained with 300 nM DAPI to visualize DNA. Arrows in the bacterial image indicated chromatin that is condensed and fragmented. Inset shows a 2.5X additional magnification of a representative bacterium from each image. *B*) High molecular weight DNA fragments were induced by HAMLET in A549 carcinoma cells (Tumor cells) and *S. pneumoniae* D39 cells and detected after 6 h and 1 h of incubation, respectively. The concentration of HAMLET used for each lane is presented in µg/ml. Increasing concentrations of HAMLET resulted in accumulation of DNA fragments over time. Low molecular weight oligonucleosomal DNA fragments were not observed in either tumor cells or bacteria treated with HAMLET (lower panel).

HAMLET-treated tumor cells displayed typical apoptotic morphology with nuclear fragmentation and chromatin condensation (Fig. 3A). A similar change in DNA morphology was observed in pneumococci as well. All pneumococci treated with HAMLET displayed condensed chromatin, seen as a reduction of the DNA volume. Associated with the DNA condensation, the chromatin was no longer homogenously stained in the bacterial cells but displayed fragmentation resulting in a punctate or patched staining pattern (Fig. 3A, see arrows).

Treatment of A549 cells with increasing concentrations of HAMLET also induced the accumulation of apoptosis-associated high molecular weight DNA fragments in the 600, 300 and 50 kbp ranges (Fig. 3B). No oligonucleosomal fragmentation was detected, however, consistent with cell death in response to HAMLET being caspase-independent [16] (Fig. 3B). High molecular weight DNA fragmentation with similar fragment sizes was also detected in pneumococci, but as with the A549 cells, this fragmentation did not proceed to oligonucleosomal fragment sizes (Fig. 3B). DNA fragmentation was not detected in pneumococci treated with detergents such as deoxycholate or SDS, suggesting that HAMLET activates specific mechanisms responsible for executing the DNA fragmentation.

**Figure 4:**
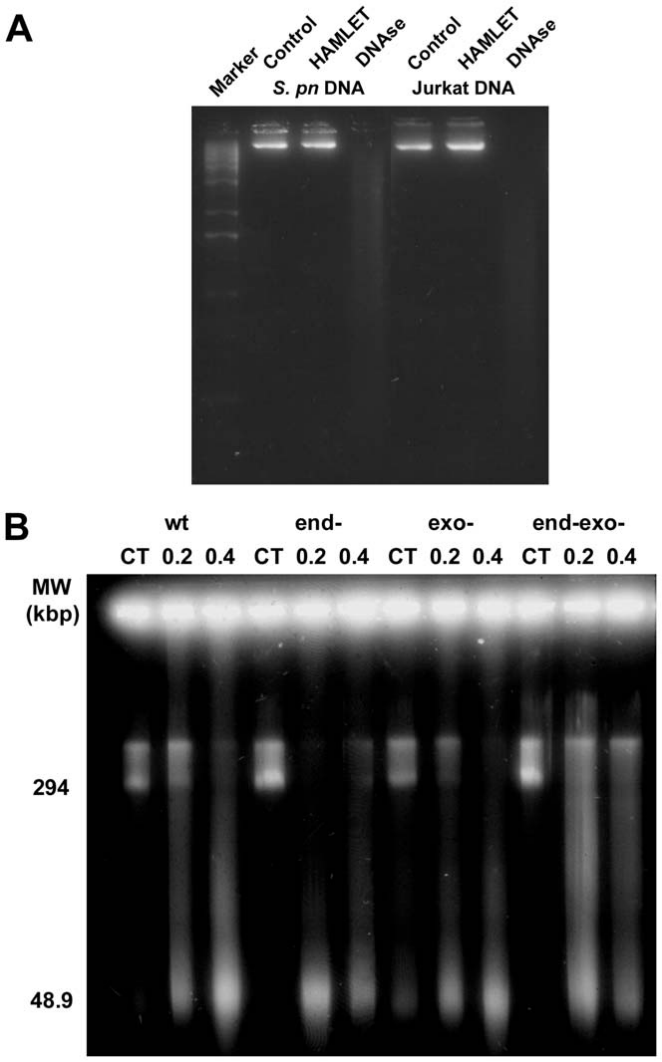
Nuclease activity in HAMLET induced DNA fragmentation. *A*) HAMLET was mixed with either pneumococcal chromosomal DNA or chromosomal DNA from Jurkat cells in the presence of both 1 mM each of Ca^2+^ and Mg^2+^ and the mixture was incubated for 1 hour and digestion was examined by gel electrophoresis. Bovine pancreas DNAse I was used as a positive control for DNA cleavage. HAMLET was unable to cleave either DNA preparation. (*B*) *S. pneumoniae* R6 (WT) and the isogenic strains 577 (**Δ** *endA*), 641 (**Δ** *exoA*) and 642 (**Δ** *endA,* **Δ** *exoA*) were incubated with 100 or 200 µg/ml of HAMLET for 2 h and high molecular weight DNA fragmentation was examined. All strains displayed an accumulation of HMW DNA fragments.

The mechanism of DNA processing in response to HAMLET has not been determined, but likely involves the nucleases that cleave DNA during apoptosis [17]. In an attempt to identify the factors responsible for cleaving the bacterial chromatin we first investigated whether HAMLET itself had DNAse activity. HAMLET was incubated with purified bacterial or tumor cell DNA in the presence of the DNAse cofactors calcium and magnesium and DNA processing was detected after separation of DNA by gel electrophoresis (Fig. 4A). HAMLET had no DNAse activity under these conditions and failed to cleave either bacterial or tumor cell DNA. DNAse I from bovine pancreas was used as a positive control and effectively processed both DNA samples (Fig. 4A).

Next we assessed the role of pneumococcal endonucleases in HAMLET-induced DNA fragmentation. The *S. pneumoniae* genome contains several open reading frames with potential nuclease activity [18]. Two nucleases necessary for uptake of DNA during genetic transformation (EndA and ExoA) have been thoroughly characterized [19]. Pneumococcal strains lacking either EndA or ExoA or a double mutant lacking both enzymes were treated with HAMLET and bacterial viability and DNA fragmentation was investigated (Fig. 4B). No difference was detected, suggesting that these nucleases were not involved in HAMLET-induced DNA fragmentation.

### 3. HAMLET induces a calcium-dependent depolarization of the mitochondrial and bacterial membranes

HAMLET targets mitochondria in tumor cells [20], [21]. In view of the common origin of bacteria and mitochondria [22], we investigated if HAMLET-treated bacteria and mitochondria might undergo similar, apoptosis-like biochemical changes.

**Figure 5:**
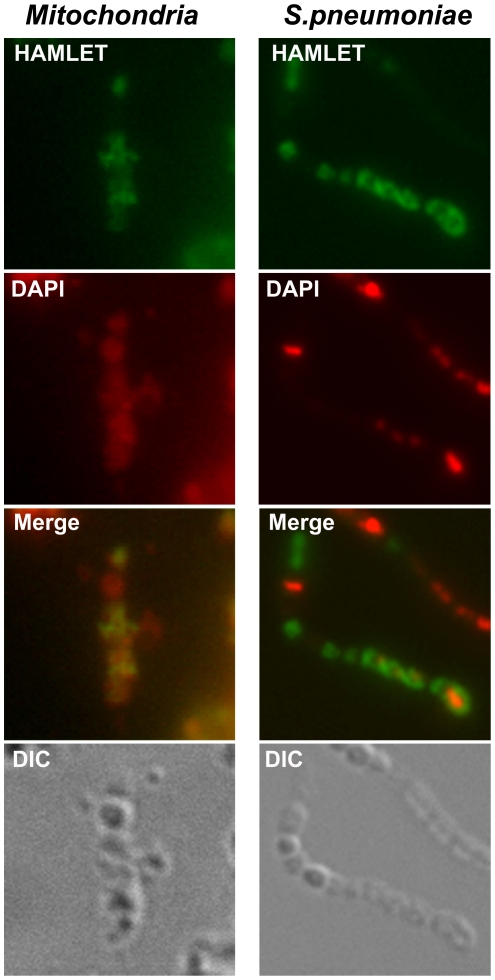
Association of HAMLET with mitochondria and pneumococci. Confocal micrographs of mitochondria (left) and *S. pneumoniae* D39 (right), incubated with a cytotoxic concentration of Alexa Fluor 488-conjugated HAMLET (100 µg/ml, green) for 1 hour at 37°C, and counterstained with DAPI (300 nM, pseudo-stained red). A light microscopy image (DIC) of each section is included (bottom panels). HAMLET associated with both bacteria and isolated mitochondria.

To compare the binding of HAMLET to mitochondria and *S. pneumoniae,* Alexa Fluor 488™-conjugated HAMLET was incubated with isolated rat liver mitochondria or with *S. pneumoniae* D39 for 30 min at 37°C. Binding was quantified by flow cytometry and visualized by confocal microscopy (Fig. 5). HAMLET bound strongly to isolated mitochondria in a dose-dependent manner. At a sublethal concentration of HAMLET (25 µg/ml), mitochondrial fluorescence was 5.5±0.3 times above the fluorescence of untreated mitochondria, detected by flow cytometry (*P*<0.01). At a lethal HAMLET concentration (100 µg/ml) the fluorescence increased to 20.2±0.5 times above untreated mitochondria (*P*<0.01). By confocal microscopy, binding to mitochondria was clearly detected but the sub-organelle distribution could not be determined due to limits of resolution (Fig. 5).

HAMLET's association with pneumococci was less pronounced. At the sublethal concentration (25 µg/ml) the bacterial fluorescence was 1.7±0.2 times higher than in untreated pneumococci, as quantified by flow cytometry (*P*<0.05). At the lethal concentration (100 µg/ml) the fluorescence increased to a mean of 5.5±0.5 times that of untreated bacteria (*P*<0.01). At this concentration, all bacteria had bound HAMLET, with one population of bacteria showing intense HAMLET-staining (Fig. 5). This population also stained with propidium iodide, a cell membrane impermeable DNA stain, indicating that these bacteria had ruptured membranes.

**Figure 6:**
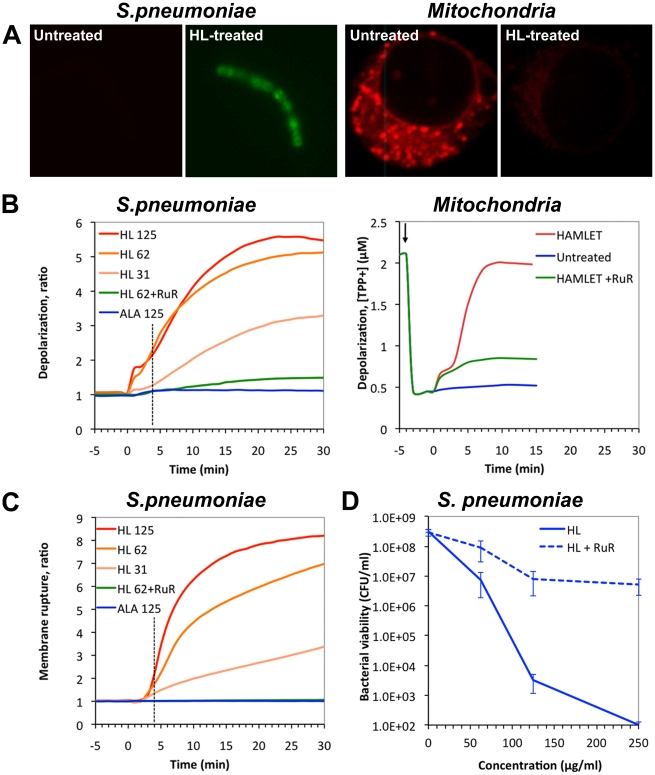
Membrane depolarization and death induced by HAMLET in mitochondria and pneumococci. *A*) Visualization of the membrane potential after HAMLET-treatment of bacteria and tumor cells for 30 minutes. Confocal micrographs depict untreated (left) and HAMLET-treated (right) *S. pneumoniae* AL2 bacteria (D39 **Δ** *lytA*) and A549 carcinoma cells. Bacterial membrane potential was visualized using the anionic bis-oxonol dye DiBAC_4_(3) that accumulates in depolarized bacteria and the tumor cell mitochondrial potential was visualized with the cationic dye TMRE that dissipates from depolarized mitochondria. Treatment of the cells with HAMLET resulted in dissipation of both the bacterial and mitochondrial membrane potential in tumor cells seen by an increased staining with DiBAC_4_(3) and a decreased staining with TMRE, respectively. *B*) Membrane potential and *C*) membrane rupture measurements in *S. pneumoniae* AL2 (D39 **Δ** *lytA*), or in isolated rat liver mitochondria. Bacterial membrane potential was monitored by the DiBAC_4_(3) and membrane rupture by an influx of propidium iodide, after treatment with 31 (HL31), 62 (HL62), or 125 (HL125) µg/ml HAMLET or 125 µg/ml ALA in the absence or presence of 30 µM Ruthenium Red (RuR). Each experiment was repeated six times and the data represents the mean ratio of the six experiments. Membrane potential in isolated mitochondria was measured by the distribution of TPP^+^ ions in the suspension in the presence of 40 nmoles of Ca^2+^ per mg protein after treatment with 50 µg/ml HAMLET in the presence or absence of 10 µM RuR. Arrow indicates addition of mitochondria. The experiment was repeated three times. The graph represents one of the three traces obtained. *D)* Effect of calcium transport inhibition on HAMLET-induced pneumococcal death. *S. pneumoniae* D39 was incubated with increasing concentrations of HAMLET in the presence or absence of 30 µM Ruthenium Red (hatched lines) and viability was monitored after 1 h of incubation by viable plate counts after overnight culture. Viability of bacteria is presented as colony forming units (CFUs) per ml suspension (detection limit in the assay was 10^2^ CFU/ml). Ruthenium Red significantly reduced HAMLETs bactericidal activity. The data represent the mean of four individual experiments with standard deviation error bars.

In previous studies, HAMLET was shown to cause mitochondrial depolarization, followed by mitochondrial swelling, permeability transition, and release of apoptogenic factors to the cytosol, resulting in activation of the cell death program [20]. To further compare the response to HAMLET between mitochondria and bacteria, depolarization of the mitochondrial membrane potential was quantified as the loss of staining with the membrane potential-sensitive dye TMRE or, using the distribution of tetraphenyl-phosphonium ion. In whole tumor cells, mitochondrial depolarization was detected as a decrease in TMRE staining within the first 30 min of HAMLET exposure (Fig. 6A). Using isolated mitochondria, binding of HAMLET was followed by dissipation of the mitochondrial membrane potential, with complete depolarization detected after approximately 10 minutes (Fig. 6B) [20]. To examine if the loss of membrane potential required Ca^2+^ the mitochondria were preincubated with the Ca^2+^-transport inhibitor Ruthenium Red and exposed to HAMLET. The response was markedly reduced (78%, *P*<0.01), suggesting that HAMLET-induced mitochondrial membrane depolarization requires Ca^2+^-transport (Fig. 6B).

Depolarization of bacterial cell membranes was detected by microscopy as an accumulation of the fluorescent dye DiBAC_4_ (3) (Fig. 6A). By fluorometry, the rapid and dose-dependent loss of membrane potential was confirmed (Fig. 6B), with kinetics similar to isolated mitochondria, as described above. Depolarization was followed by membrane rupture detected by an influx of propidium iodide starting approximately 3–5 minutes after the addition of HAMLET (Fig. 6C). The dose-dependent depolarization was directly associated with a dose-dependent killing (1.6, 2.8, and 5.0 log_10_ viability reduction at 31, 62, and 125 µg/ml of HAMLET after 1 hour; Fig. 6D). Similar to mitochondria, Ruthenium Red (30 µM) reduced the loss of membrane potential in *S. pneumoniae* by 88.5% (*P*<0.0001; Fig. 6B), protected against membrane rupture by 97.6% (*P*<0.0001; Fig. 6C) and reduced log-death by 50.5% (*P*<0.0001; Fig. 6D). The results suggest that HAMLET directly influences the membrane potential, in *S. pneumoniae* and mitochondria, and indicate that Ca^2+^ transport is involved in both systems.

### 4. Role of protease activity in HAMLET-induced death

Mitochondrial permeability transition leads to a release of apoptogenic factors from mitochondria, which induce apoptosis in eukaryotic cells, either with or without caspase activation [16]. Even though exposure of tumor cells to HAMLET results in cytochrome c-release from mitochondria and activation of caspases, inhibitors of these pathways do not prevent cell death and cell death is not regulated by Bcl-2 or p53 family proteins [23], [24], demonstrating that the effects of HAMLET on cell viability are independent of common apoptosis effector molecules. The pan-caspase inhibitor zVAD-fmk also does not inhibit high molecular weight DNA fragmentation in response to HAMLET [23].

**Figure 7:**
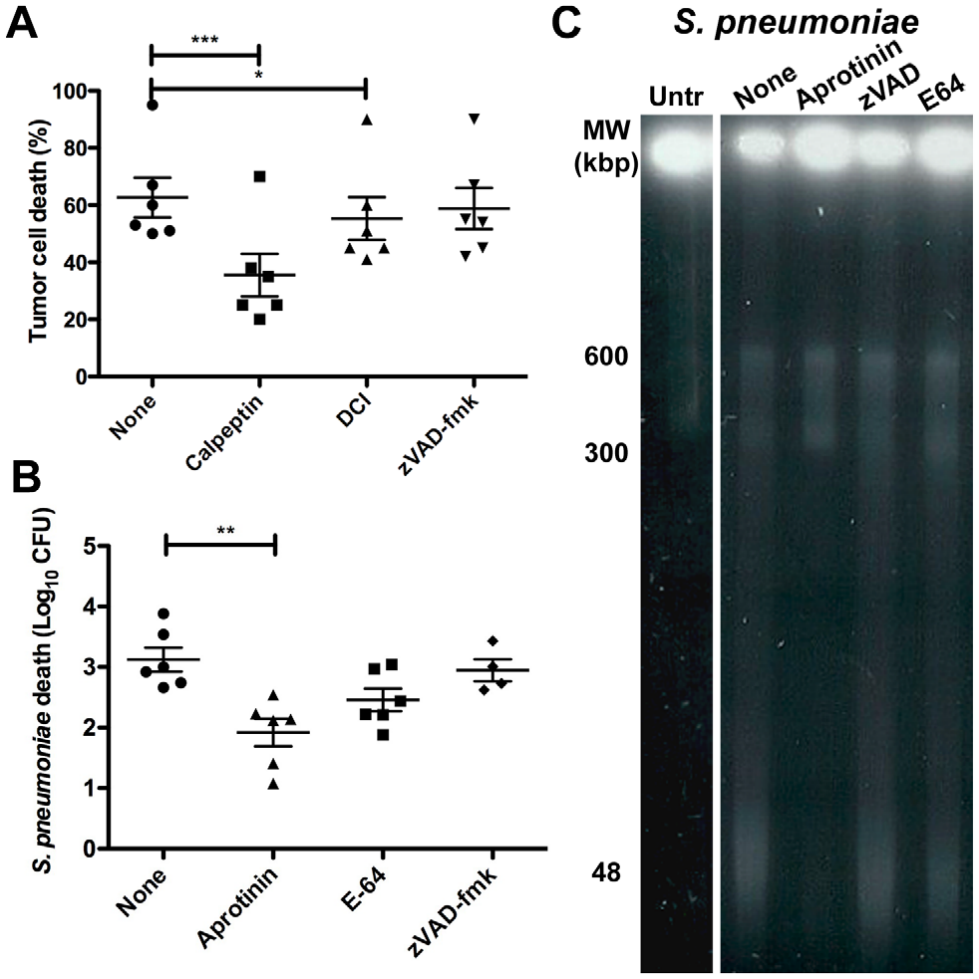
Role of serine proteases in HAMLET-induced death of tumor cells and pneumococci. A) A549 carcinoma cells were preincubated for 10 minutes with diluent, 25 µM zVAD-fmk (pan-caspase inhibitor), 100 µM dichloroisocoumarin (serine protease inhibitor), or 100 µM calpeptin (calcium-dependent cysteine protease inhibitor) before being treated with 300 µg/ml of HAMLET. After 6 hours of incubation cell viability was measured using trypan blue exclusion. The graph depicts the mean death in % obtained after 3 individual experiments. The error bars represent the standard deviation. * and *** represent *P*<0.05 and *P*<0.001, respectively. B and C) *S. pneumoniae* AL2 (D39 **Δ** *lytA*) were preincubated for 10 minutes with diluent, 25 µM Aprotinin (serine protease inhibitor), 25 µM zVAD-fmk (pan-caspase inhibitor), or 10 µM E-64 (cysteine protease inhibitor) before being treated with 50 µg/ml of HAMLET. After 2 hours viability was determined and samples were analyzed for high molecular weight DNA fragmentation. *B)* Viability. The graph depicts the mean log_10_ death obtained from five individual experiments. The error bars represent the standard deviation. ** represents *P*<0.01. C) DNA fragmentation. Untr indicates untreated bacteria. The remaining samples were treated with HAMLET in the presence of diluent (none) or proteasee inhibitors. Only the serine protease inhibitors aprotinin rescued pneumococci from death and DNA fragmentation.

To address the role of other proteases, tumor cells were preincubated with the calcium-dependent cysteine protease inhibitor calpeptin or the serine protease inhibitor dichloroisocoumarin (DCI) and effects on HAMLET-induced tumor cell death and DNA fragmentation were recorded (Fig. 7A). Calpeptin inhibited HAMLET-induced log-death by 43% (*P*<0.001) and effectively blocked DNA fragmentation in response to HAMLET. DCI significantly reduced death in response to HAMLET (12%; *P*<0.05) and almost completely blocked DNA fragmentation.

To evaluate the role of proteases in bacterial death we first searched the pneumococcal genome for caspase-homologues, but no none were identified. To address if bacteria express other proteases with caspase-like activity, *S. pneumoniae* D39 lysates were incubated with the three major groups of caspase-substrates YVAD-, IETD-, and DEVD-amc in a fluorescence-based assay but no activity was detected. To verify the lack of caspase-related activity, bacteria were incubated with the pan-caspase inhibitor zVAD-fmk or with the cysteine protease inhibitor E-64. No significant effect on bacterial viability was observed and DNA fragmentation remained unchanged (Fig. 7B and C). In contrast, pretreatment with the serine protease inhibitor aprotinin reduced HAMLET-induced log-death by 39% (Fig. 7B, *P*<0.01) and inhibited DNA fragmentation (Fig. 7C), indicating a role for this family of proteases in the execution of HAMLET-induced bacterial death.

### 5. Effect of HAMLET on other bacterial species

**Figure 8:**
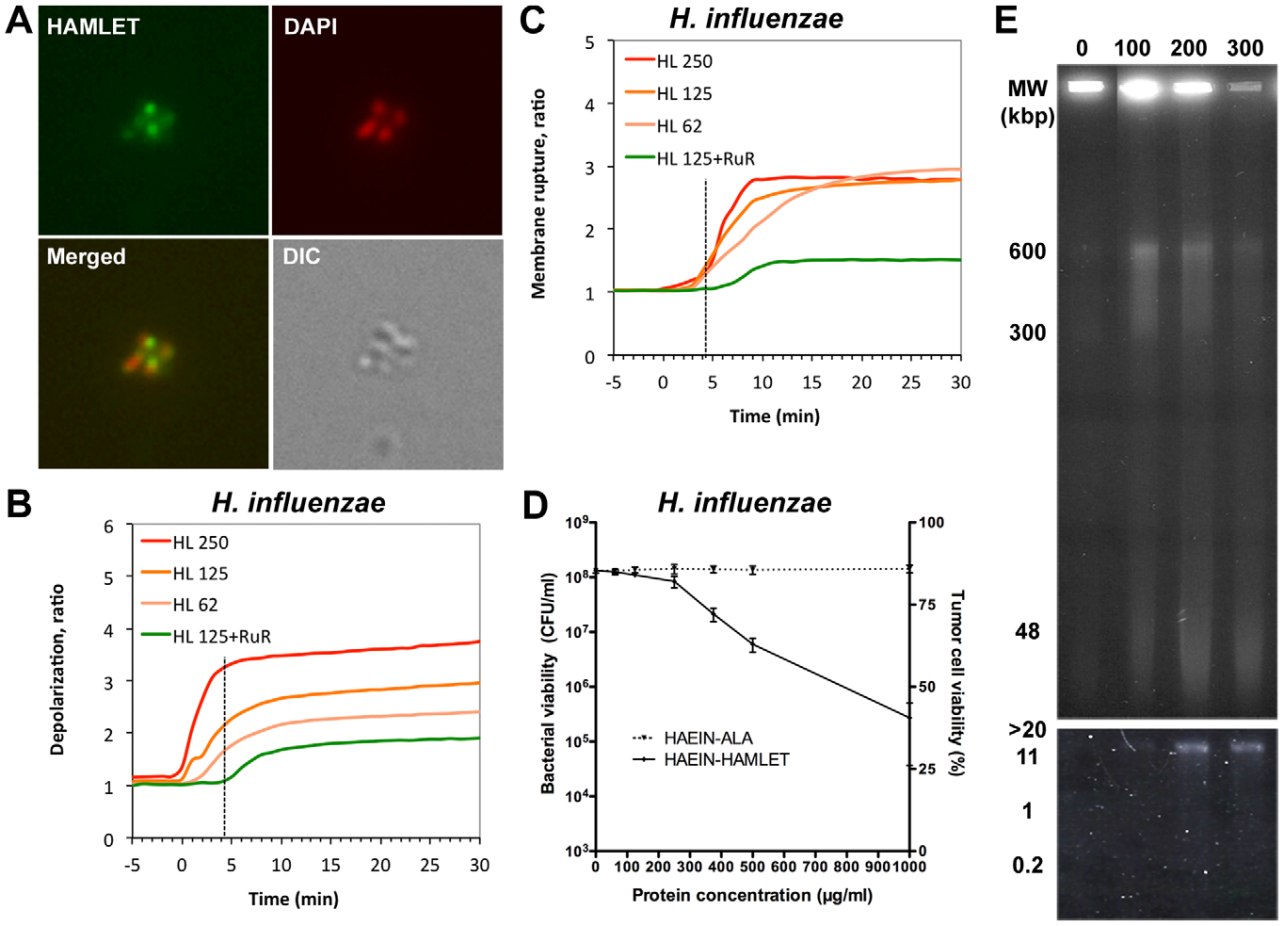
Apoptosis-like changes in HAMLET-treated *H. influenzae*. A) Association of HAMLET with bacteria. Confocal micrographs of *H. influenzae* 2019, incubated with a cytotoxic concentration of Alexa Fluor 488-conjugated HAMLET (250 µg/ml, green) for 1 hour at 37°C, and counterstained with DAPI (300 nM, pseudo-stained red). A light microscopy image (DIC) of each section is included in the bottom row. *B*) Membrane potential and *C*) membrane rupture measurements in *H. influenzae* 2019. Bacterial membrane potential was monitored by the DiBAC_4_(3) and membrane rupture by an influx of propidium iodide, after treatment with 62 (HL62), 125 (HL125), or 250 (HL250) µg/ml HAMLET or 250 µg/ml ALA in the absence or presence of 30 µM Ruthenium Red (RuR). Each experiment was repeated six times and the data represents the mean ratio of the six experiments. *D)* Effect of calcium transport inhibition on HAMLET-induced pneumococcal death. *H. influenzae* 2019 was incubated with increasing concentrations of HAMLET in the presence or absence of 30 µM Ruthenium Red (hatched lines) and viability was monitored after 1 h of incubation by viable plate counts after overnight culture. Viability of bacteria is presented as colony forming units (CFUs) per ml suspension (detection limit in the assay was 10^2^ CFU/ml, (mean of four experiments with standard deviation error bars). E) Chromatin fragmentation induced by HAMLET in *H. influenzae*. High molecular weight DNA fragments were induced by HAMLET in *H. influenzae* 2019 cells and detected after 1 h of incubation. (HAMLET concentration in µg/ml). Increasing concentrations of HAMLET resulted in accumulation of DNA fragments over time. Low molecular weight oligonucleosomal DNA fragments were not observed (lower panel).

In addition to *S. pneumoniae*, HAMLET effectively kills other streptococcal species as well as the respiratory pathogen, *Haemophilus influenzae* [3]. This pathogen was selected to investigate if the apoptosis-like response also occurs in other bacterial species. HAMLET was found to bind to *H. influenzae* cells (Fig. 8A) and binding resulted in depolarization (Fig. 8B) of the bacterial membrane. Although the depolarization was more rapid than in pneumococci it showed less pronounced changes (2–3 fold increase in fluorescence compared to 5–6 fold increase in pneumococci). Membrane depolarization resulted in rupture of the bacterial membrane (Fig. 8C) and loss of viability (Fig. 8D), both less pronounced than for pneumococci. As for pneumococci, Ruthenium Red inhibited depolarization and death, with membrane potential being reduced by 52.8% (*P*<0.01; Fig. 8B), membrane rupture by 72.1% (*P*<0.01; Fig. 8C) and log_10_ death decreased by 62.4% (*P*<0.05; Fig. 8D).

DNA fragmentation in response to HAMLET was also detected in *H. influenzae,* with a dose-dependent accumulation of high molecular weight DNA fragments in the 600, 300 and 50 kbp ranges, similar to the DNA fragmentation which occurred in HAMLET-treated tumor cells and pneumococci (Fig. 8E). No oligonucleosomal fragmentation was detected. These results suggest that HAMLET's bactericidal mechanism is similar in different HAMLET-sensitive bacterial species and not restricted to gram-positive or gram-negative organisms.

## Discussion

Apoptotic cell death is critical for eukaryotic cell turn over, tissue development and homeostasis. Here we describe that features of apoptosis can be activated also in bacterial cells. Striking similarities were observed in cellular responses of eukaryotic and prokaryotic cells to the human milk complex HAMLET, including cell death accompanied by DNA fragmentation and a change in morphology with cell shrinkage and DNA condensation. HAMLET bound to eukaryotic and prokaryotic cell membranes and induced a calcium-dependent depolarization of the plasma- or mitochondrial membranes as well as bacterial cells, followed by downstream degradation pathways involving protease and endonuclease activity. The similarities between mitochondrial and bacterial responses to HAMLET are consistent with their shared evolutionary origin and suggest that HAMLET activates targets that are conserved in tumor cells and certain bacteria. The results support our speculation that death activation pathways evolved early, and suggest that similar mechanisms may be shared by prokaryotic and eukaryotic organisms. Characterizing these basic mechanisms of cell death regulation may be important for future disease therapies involving both eukaryotic and prokaryotic cells.

HAMLET shows a specific activity against certain bacterial species but fails to kill others [3]. *S. pneumoniae* and many other streptococci are sensitive, undergoing apoptosis like changes including DNA fragmentation, but most other species, including *Escherichia coli* and Staphylococci, are not killed. The membranes of sensitive species are rapidly depolarized by HAMLET. In a screen of bacterial HAMLET sensitivity, we observed that resistant species such as Staphylococci respond to HAMLET with low membrane depolarization and a difference in signaling threshold between sensitive and resistant bacteria is further supported by our observation that both HAMLET-sensitive and resistant species can undergo death with apoptosis-like morphology in response to other death stimuli, such as starvation. Apoptotic response pathways may thus be present in most bacteria but differentially activated depending on the agonist. The difference in HAMLET sensitivity was not related to the bacterial structure as defined by gram-positive or gram-negative staining, but it may be speculated that sensitivity has evolved to fit the niche the organisms inhabit. In the case of HAMLET, sensitive species are found primarily in the oral cavity and respiratory tract, which are exposed to human milk and its constituents during breast feeding.

HAMLET triggered high molecular weight DNA fragmentation in both carcinoma cells and bacteria but no oligonucleosomal fragments were observed. In eukaryotic cells, proteolytic cleavage of lamins and other structural DNA-associated proteins is necessary for the early formation of high molecular weight DNA fragments ranging from 50–600 kbp [25]. These DNA fragments represent excised DNA loops and oligomers that precede the oligonucleosomal DNA fragmentation [5]. The shared DNA fragmentation pattern displayed by eukaryotic and bacterial cells after HAMLET-treatment suggests that the topology and packing of the DNA may be more similar than currently appreciated.

Protease activation is commonly required for the unpacking of DNA and for endonuclease activation during eukaryote death [5], [26]. In tumor cells, HAMLET required calpain and serine protease activity to exert its effects, as death and DNA fragmentation were effectively inhibited by calpeptin and DCI, respectively. For *S. pneumoniae,* we show that HAMLET-induced DNA fragmentation was independent of the two well-characterized endonucleases EndA and ExoA but required serine proteases, as inhibition by aprotinin effectively rescued HAMLET-treated pneumococci from DNA fragmentation and death. While the exact mechanisms remain to be identified, our observations suggest that DNA processing in response to HAMLET may be a universal aspect of cell death in both eukaryotic and certain prokaryotic cell kingdoms. In pneumococci, this leaves a number of open reading frames with potential endonuclease activity, which might be involved in DNA repair and DNA catabolism [18].

HAMLET's binding to mitochondria and bacteria caused a rapid loss of membrane potential. This effect was inhibited by Ruthenium red, which blocks calcium fluxes. In eukaryotic cells, calcium signaling plays a clear and prominent role in the regulation of many cellular processes including cell death [27]–[29]. An increase in cytosolic calcium invariably stimulates mitochondrial uptake through the mitochondrial uniporter and other systems and excessive calcium uptake by mitochondria causes depolarization of the mitochondrial membrane and opening of the permeability transition pore, which leads to the release of apoptogenic factors that trigger the execution phase of apoptotic cell death [27], [30], [31]. A role for calcium in HAMLET-induced permeability transition in isolated mitochondria has previously been suggested, based on observations that EGTA inhibited HAMLET-induced swelling of mitochondria and the release of cytochrome c [20].

The role of calcium in bacterial cell signaling is more elusive [32], [33]. There is evidence for a role of calcium in responses to environmental stresses as well as a potential role in cell cycle progression and differentiation processes such as sporulation and fruiting body development, but no information is available regarding calcium's involvement in bacterial cell death [32]. While Ruthenium Red inhibited HAMLET-induced depolarization of the bacterial membrane and rescued the bacteria from death, the calcium transport mechanism induced by HAMLET remains unknown. Pneumococci express a P-type Ca^2+^-ATPase for calcium efflux and calcium transport through a sodium/calcium exchanger has been proposed to regulate competence, DNA uptake and lysis, but the transporter has not been identified and is not annotated in published genomes [34], [35]. Furthermore, BLAST analysis of both the pneumococcal and *H. influenzae* genomes against the transport classification database (http://www.tcdb.org/) failed to identify any other calcium transporters, suggesting that sequences and motifs differ from those characterized so far.

In multicellular organisms, controlled elimination of aged, faulty or potentially harmful cells is an important feature to maintain functional homeostasis of the organism, and the need for such mechanisms is easily apparent. It is especially important to degrade defective DNA to avoid the persistence of mutated DNA that could become detrimental to the organism. Bacteria may require similar mechanisms, especially since they often grow in aggregated communities or biofilms. Specialization of bacterial function (terminal differentiation) or sacrifice of some individual cells in favor of others (altruism) within these communities appears to exist and elimination of cells using a genetically inherent pathway could be advantageous [8], [36]. This is especially evident for *S. pneumoniae* where it was recently shown that the release of the intracellular virulence factor pneumolysin, as well as DNA, is due to the predation of genetically competent organisms on non-competent organisms surrounding them, a phenomenon named fratricide, which has been seen also in other bacterial species [37]. This would also indicate that the release of fragmented DNA in response to lethal stimuli can benefit the bacterial “community” by increasing the spread of genetic information, including antibiotic resistance, to surrounding bacteria and be used to form an intercellular matrix in biofilms [36]. Indeed, such DNA release may well be critical to the very efficient pneumococcal genetic transformation originally described by Avery [38]. Molecules such as HAMLET may thus have evolved to help the infant combat potentially harmful infections early in life [39], and/or to help the infant regulate cell proliferation during the early, rapid growth and development of mucosal tissues [40].

## Materials and Methods

### Reagents

DEAE-Trisacryl M was from BioSepra (Villeneuve la Garenne, France). SeaKem GTG agarose and SeaPlaque GTG Low melting temperature agarose gel and were from SeaKem, FMK Bioproducts (Rockdale, USA). Trypan blue was from Chroma Gesellschaft, Schmid & Co (Stuttgart, Germany).

### Production of HAMLET

HAMLET was produced by converting native alpha-lactalbumin in the presence of oleic acid (C18:1) as described [2]. Briefly, native alpha-lactalbumin was purified from human milk by ammonium sulfate precipitation and phenyl sepharose chromatography [41]. Apo alpha-lactalbumin was generated from 25 mg of native alpha-lactalbumin dissolved in Tris (10 mM Tris/HCl pH 8.5) by addition of 3.5 mM EDTA. Conversion of apo-alpha-lactalbumin to HAMLET was achieved by ion exchange chromatography on DEAE-Trisacryl M matrix conditioned by addition of 10 mg of C18:1 fatty acid. The protein was eluted by applying increased concentrations of NaCl in a Tris buffer devoid of EDTA.

### Cells

Jurkat leukemia cells were obtained from the European Collection of Cell Cultures (Wiltshire, UK) and A549 cells (CCL-185) were obtained from the American Type Culture Collection (Manassas, VA, USA). Both cell types were cultured in RPMI 1640 medium supplemented with 10% fetal calf serum, 2 mM glutamine, non essential amino acids, sodium pyruvate, and 50 µl gentamicin/ml, at 37°C in a humidified atmosphere containing 5% CO_2_. The effect of HAMLET and ALA on cell viability was assessed by measuring the exclusion of trypan blue (Invitrogen) in the cell population.

### Bacteria

The following isolates of *S. pneumoniae* were used in the study: DBL5 [42], WU2 [43], TIGR4 [18], L82006, L81905, L82016, L82013, BG9273, BG7322, BG30-11, BG8826 [44], EF10197, EF10175, EF3030, EF3296, EF3559, EF1488 [45], [46], ATCC 6303 [47], 3JYP2670 [48], ATCC 46919 [49] A66, and D39 [38]. Furthermore D39 pneumococci lacking the autolysin LytA, AL2 [50], PspA [51], PspC [52], PsaA [53], pneumolysin [54], and DLDH [55] were used to evaluate the role of virulence factors for HAMLET-induced pneumococcal death. These mutants were all produced through insertion duplication mutagenesis where the target gene was interrupted with a plasmid carrying erythromycin resistance. Strains 577, 641, and 642, lacking nucleases were kindly provided by Dr Sanford Lacks, New York, USA [56], [57]. *H. influenzae* strain 2019 was kindly provided by Dr Campagnari, University at Buffalo, SUNY [58]. *H. influenzae* strain Eagan (type b) [59] and Rd (type e) [60] were kindly provided by Jeffrey Weiser, University of Pennsylvania, Philadelphia, PA.

The pneumococcal strains were stored in glycerol stocks at −80°C, and frozen stocks were used to seed Todd Hewitt medium containing 0.5% Bacto-Yeast extract. *Haemophilus influenzae* were grown on chocolate agar and seeded into Brain heart infusion broth containing 10 ml/L IsoVitaleX enrichment solution (BD Biosciences), 5% fetal bovine serum (Invitrogen), and 25 mg/L hemin (Sigma). In late logarithmic growth phase, the bacteria were harvested by centrifugation at 1200× *g* for 20 minutes and suspended in phosphate-buffered saline (PBS; 30 mM Na_2_HPO_4_, 10 mM KH_2_PO_4_, 120 mM NaCl, pH 7.4). Appropriate dilutions of the bacteria were suspended in PBS. The effect of HAMLET on bacterial viability was assessed by viable counts on blood agar or chocolate agar plates, respectively.

### Chromatin condensation and fragmentation

For detection of high molecular weight DNA fragments, treated bacteria and cells were pipetted into gel plugs that were treated with proteinase K for 24 hours as described [1]. Gel electrophoresis was run at 12°C, 175 V in 1% agarose gels in 0.5× TBE (45 mM Tris, 1.25 mM EDTA, 45 mM boric acid, pH 8.0), with the ramping rate changing from 0.8 seconds to 30 seconds for 24 hours, using a forward to reverse ratio of 3:1. DNA fragment size was calibrated using two sets of pulse markers: chromosomes from *Saccharomyces cerevisiae* (225–2200 kbp) and a mixture of lDNA Hind III fragments, lDNA and lDNA concatemers (0.1–200 kbp) from Sigma.

To study DNA morphology of bacteria or cells, treated bacteria were fixed in 4% paraformaldehyde and exposed to 300 nM DAPI (Invitrogen) and viewed by fluorescence microscopy using a Leica DMI6000 microscope (Leica Microsystems, Bannockburn, IL).

### Isolation of mitochondria

Jurkat cells were pelleted and washed in buffer, containing 100 mM sucrose, 1 mM EGTA and 20 mM MOPS, resuspended in 5% Percoll, 0.01% digitonin and protease inhibitors and incubated on ice for 10 min, followed by centrifugation at 2,500× g for 5 minutes. The supernatant was subjected to an additional centrifugation at 10,000× g for 15 minutes, mitochondrial pellet was collected in 300 mM sucrose, 1 mM EGTA, 20 mM MOPS and protease inhibitors and kept at −70°C. Mitochondria (3 mg/ml) were transferred into buffer, containing 250 mM sucrose, 10 mM MOPS, 5 mM succinate, 3 mM KH_2_PO_4_, 10 µM EGTA and 10 mM Tris, pH 7.5 and after incubation at 30°C centrifuged at 10,000× g for 15 minutes.

### Binding of HAMLET to mitochondria and bacteria

HAMLET protein was directly labelled with Alexa Fluor™ 488 (Molecular Probes Inc) according to manufacturer's instructions. Bacteria (10^8^/ml, 100 µl) or mitochondria (3 mg/ml protein concentration, 25 µl) were incubated with fluorescently labelled HAMLET at 37°C for various 30 min. The fluorescence intensity of the bacteria were analyzed in a FACSCalibur flow cytometer (BD) using a 520 nm band-pass filter or bacteria and mitochondria were counterstained with 300 nM DAPI and inspected by confocal microscopy.

### Measurement of membrane potential

For a visual depiction of the membrane potential in whole cells, Jurkat cells were treated with 0.3 mg/ml of HAMLET for 30 min and 25 nM of TMRE was added 15 minutes before the cells were inspected by confocal microscopy. The potential over the mitochondrial membrane of isolated mitochondria was measured using an electrode sensitive to the cation tetraphenylphosphonium as described [20].

Bacteria were pelleted by centrifugation at 2,400× *g* and washed twice by resuspension in PBS (pH 7.2) followed by centrifugation. The bacterial pellet was resuspended in half the original volume of PBS and energized with 50 mM glucose for 15 min at 37°C. To energized bacteria, propidium iodide (40 µg/ml) and DiBAC_4_(3) (0.5 µM) were added and 100 µl bacterial suspension was added to 100 µl of PBS, HAMLET or ALA in each well of a 96-well microtiter plate (Falcon, BD Biosciences) resulting in a final concentration of 25 mM glucose, 20 µg/ml PI and 250 nM DiBAC_4_(3) in each well. The plate was immediately placed into a 37°C pre-warmed Synergy II microplate reader (Biotek, Winooski, VT) and the DiBAC_4_(3) fluorescence (485/20 nm excitation, 520/25 nm emission) and PI fluorescence (485/20 nm excitation, 590/35 nm emission) were read every minute for 60 minutes to monitor the change membrane polarity and integrity, respectively.

### Protease activity assays

Bacterial extracts were produced as described above. Aliquots containing bacterial extract were then transferred to a 96-well plate and 50 µl of freshly prepared substrate buffer (100 mM HEPES, 10% sucrose, 0.1% CHAPS, 5 mM DTT 10^−6^% NP-40, pH 7.25) containing either of the substrates Ac-YVAD-amc, Ac-DEVD-amc or Ac-IETD-amc was added per well. The final concentration of Ac-YVAD-amc, Ac-DEVD-amc or Ac-IETD-amc was 14.4 µM, 33.3 µM and 13.6 µM, respectively. The enzymatic reaction was carried out at 37°C and the rates of hydrolysis were measured by release of amc from the substrates using an ELISA reader. Experiments were performed in duplicates and the activity was expressed as change in fluorescence units per min per 10^6^ cells.

For protease inhibition studies, *S. pneumoniae* AL2 (D39 **Δ** *lytA*) were preincubated for 10 minutes in the presence of diluent, 25 µM aprotinin, 10 µM E-64, or 25 µM ZVAD-fmk before being treated with 50 µg/ml of HAMLET for 2 hours. Bacterial cells were then diluted and plated for determination of colony forming units on blood agar after overnight culture.

### Nuclease activity assays

Nuclease activity of HAMLET was tested as the ability to cleave chromosomal DNA from D39 bacteria or Jurkat cells. Chromosomal DNA from cells and bacteria was prepared by standard procedures using phenol/chloroform extraction.

For tests of nuclease activity 1 µg of *S. pneumoniae*, or Jurkat chromosomal DNA were mixed with 50 µg/ml HAMLET in phosphate buffered saline (30 mM Na_2_HPO_4_, 10 mM KH_2_PO_4_, 120 mM NaCl, pH 7.4) with 1 mM Ca^2+^ and 1 mM Mg^2+^, and incubated for 1 hour at 37°C, and run by agarose gel electrophoresis in 1.5% gels with a constant voltage of 100 V. DNAse I from bovine pancreas (100 Kuntz units/ml) was used as a positive control.

### Statistical analysis

Quantitative data were analysed from a minimum of three repeats using Student's T-test with a 2-tailed *P*-value. The *n* for each analysis is presented in the figure legends.
